# Stochastic Model of In-Vivo X4 Emergence during HIV Infection: Implications for the CCR5 Inhibitor Maraviroc

**DOI:** 10.1371/journal.pone.0038755

**Published:** 2012-07-17

**Authors:** Borislav Savkovic, Geoff Symonds, John M. Murray

**Affiliations:** 1 School of Mathematics and Statistics, University of New South Wales, Sydney, Australia; 2 St. Vincent's Centre for Applied Medical Research, Darlinghurst, New South Wales, Sydney, Australia; 3 Kirby Institute, University of New South Wales, Sydney, Australia; New York University, United States of America

## Abstract

The emergence of X4 tropic viral strains throughout the course of HIV infection is associated with poorer prognostic outcomes and faster progressions to AIDS than for patients in whom R5 viral strains predominate. Here we investigate a stochastic model to account for the emergence of X4 virus via mutational intermediates of lower fitness that exhibit dual/mixed (D/M) tropism, and employ the model to investigate whether the administration of CCR5 blockers in-vivo is likely to promote a shift towards X4 tropism. We show that the proposed stochastic model can account for X4 emergence with a median time of approximately 4 years post-infection as a result of: 1.) random stochastic mutations in the V3 region of env during the reverse transcription step of infection; 2.) increasing numbers of CXCR4-expressing activated naive CD4+ T cells with declining total CD4+ T cell counts, thereby providing increased numbers of activated target cells for productive infection by X4 virus. Our model indicates that administration of the CCR5 blocker maraviroc does not promote a shift towards X4 tropism, assuming sufficient efficacy of background therapy (BT). However our modelling also indicates that administration of maraviroc as a monotherapy or with BT of suboptimal efficacy can promote emergence of X4 tropic virus, resulting in accelerated progression to AIDS. Taken together, our results demonstrate that maraviroc is safe and effective if co-administered with sufficiently potent BT, but that suboptimal BT may promote X4 emergence and accelerated progression to AIDS. These results underscore the clinical importance for careful selection of BT when CCR5 blockers are administered in-vivo.

## Introduction

CCR5 blockers are a promising new class of anti-HIV drugs that act by binding to the CCR5 coreceptor, thereby reducing the number of CD4-CCR5 complexes available for viral binding by HIV and consequently inhibiting the viral entry stage of the infection cycle [1]. Recent evidence has shown that individuals carrying a Δ-32 mutation have reduced CCR5 expression on the surface of their CD4+ T cells, thereby achieving full (homozygous) or partial (heterozygous) protection against HIV due to decreased likelihood of viral entry [2], [3], [4], [5]. This and the “curative effect” seen from transplantation of Δ-32 mutation hematopoietic stem cells to the Berlin patient with AIDS and leukemia [6] have given strong impetus for the use of entry-inhibitors for HIV. The importance of inhibiting viral entry is further emphasised by recent reports that over 95% of HIV-induced cell death is attributable to bystander apoptosis resulting from viral entry into a cell without viral integration into the cellular genome [7]. Recent trials of the CCR5 entry-inhibitor maraviroc reported promising clinical outcomes [8], [9], [10], [11], [12], with maraviroc administered with Optimized Background Therapy (OBT) achieving significantly higher increases in CD4+ T cell counts over the duration of the study than placebo (OBT only).

A key concern with the administration of CCR5 blockers in-vivo relates to the emergence of CXCR4 (X4) tropic virus [1], which is associated with worse clinical outcomes than CCR5 (R5) tropic virus and with faster clinical progression to AIDS [13], [14], [15]. X4 virus also appears more pathogenic and virulent than R5 [14], [16], [17]. Reports of three macaques dually-infected with R5 and X4 tropic SIV reported increased X4 tropic viral loads following administration of the CCR5 blocker CMPD 167 [18]. Furthermore, recent trials of maraviroc reported emergence of dual/mixed (D/M) viral strains following administration of therapy (maraviroc+OBT) in patients in whom no X4-tropism could be detected prior to the administration of therapy [8], [9], [10], [11], [12]. More detailed clonal analysis of these patients however reported the increased D/M tropism to be attributable to outgrowth of pre-existing and previously undetected minority populations of CXCR4-using virus [19]. These observations of increased X4-tropism emphasise the need for an increased quantitative understanding of the selective pressures governing X4 emergence in-vivo when CCR5 blockers are administered.

Over the course of untreated infection, X4 tropic virus generally emerges at later stages of infection [13], [14], [15], although X4 viral strains have also been reported at early stages [20]. Although the reasons behind in-vivo X4 emergence remain unknown, a recent line of evidence indicates that X4 emergence might be driven by changes in the host environment, resulting in increasing numbers of activated naive CD4+ T cells (CD4+HLA-DR+CD45RA+) at later stages of the infection when total CD4+ T cell numbers are low [21]. This provides for increased numbers of activated CD4+ T cells for productive infection by X4 virus, but not R5 virus, since activated naive CD4+ T cells exhibit high expression of the CXCR4 chemokine receptor on the cell surface, with no/negligible CCR5 expression [22]. In contrast, activated memory CD4+ T cells (CD4+HLA-DR+CD45RO+), which constitute the majority of activated CD4+ T cells at early stages of the infection, predominantly express CCR5,with relatively lower per-cell density of CXCR4 [21], [22], [23], [24]. Within this target-cell activation hypothesis, the predominance of R5 virus at early stages of the infection [21], [25] is thus attributable to the majority of activated CD4+ T cells at early stages expressing a memory phenotype.

In the present study we investigate a *stochastic* model to account for X4 emergence in-vivo. While previous *deterministic* modelling of the X4 switch demonstrated that X4 emergence can in principle be accounted for by increased activation of naive CD4+ T cells at later stages of infection [21], [23], [24], in the present analysis we extend these results by modelling X4 emergence as a *stochastic* process. We assume that the tropism shift is also driven by random viral mutations during the reverse transcription step of the infection cycle [26]. In particular, mutations in the V3 region of the env gene determine viral tropism [27], [28], [29], [30], [31], and we assume that mutation from R5 to X4 virus occurs via D/M intermediates of reduced fitness [32]. Since X4 emergence is a *stochastic* event, with a median emergence time of approximately 4 years post-infection with considerable variation around this time [15], the model presented here is more likely to be clinically relevant than previously presented *deterministic* models.

Our model is employed to investigate the course of HIV infection when maraviroc is administered at early, intermediate or late stages of infection, and also when co-administered with background therapy (BT) of variable efficacies. Previous modelling has only considered the administration of therapy at a single time-point [21], [23], [24]. Our results indicate that application of maraviroc is safe at any stage of infection if administered with sufficiently potent BT. However our results also indicate that maraviroc can promote X4 emergence and accelerated progression to AIDS when administered as a monotherapy or when administered with BT of suboptimal efficacy. These results highlight the importance for careful BT selection when CCR5 blockers are administered in-vivo.

## Methods

### Model Equations

**Figure 1:**
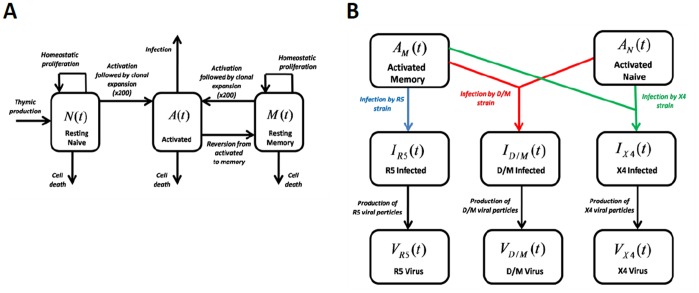
Mathematical model. Shown are the model compartments. A) Compartment model of the uninfected CD4+ T cell subpopulations. Here 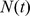, 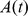 and 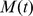 denote resting naive, activated and resting memory CD4+ T cells respectively; B) Compartment model of activated cells, productively infected cells and of free virus. Here 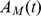 denotes activated memory CD4+ cells (CD4+HLA-DR+CD45RO+) that predominately express CCR5 but also express CXCR4, whereas 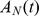 denotes activated naive CD4+ T cells (CD4+HLA-DR+CD45RA+) that express CXCR4 with no/negligible CCR5 expression. The total number of activated cells (CD4+HLA-DR+) is given by 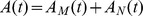. R5-tropic virus is only assumed to infect activated memory CD4+ T cells. Dual/mixed (D/M) and X4 tropic strains are assumed to infect both activated memory as well as activated naive CD4+ T cells. Here 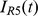, 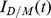 and 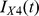 denote productively infected cells of strains R5, D/M and X4 respectively. 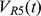, 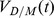 and 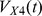 denote the number of viral particles of strains R5, D/M and X4 respectively. In our model, infection of an uninfected cell by one strain may result in productively infected cells of a different strain, as a result of random mutations during the reverse transcription step of the infection cycle (not shown in figure; see Methods).

Three uninfected CD4+ T cell subpopulations are modelled (Figure 1A): resting naive, activated and resting memory CD4+ T cells. The model parameters are given in Table 1 and in Table 2. The equations governing uninfected CD4+ T cell dynamics, the number of productively infected cells and viral dynamics (Figure 1 A,B) are given by the following set of differential equations:

**Table 1:** Table of model parameters relating to dynamics of uninfected CD4+ T cell populations.

Parameter	Parameter description	Parameter value	Source
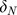	Death rate of resting naive cells	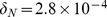 1/day	From [67].
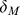	Death rate of resting memory cells	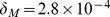 1/day	From [67].
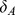	Death rate of activated cells	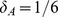 1/day	From [68]. Implies 5 day half-life for activated CD4+ T cell.
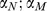	Normal activation rates of resting naive ( 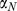 ) and resting memory cells ( 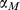 )	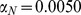 1/day; 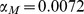 1/day	Determined by calibration to give constant total CD4+ T cell levels of approximately 1000 cells/µL in PB in a healthy individual, with 500 cells/µL memory and 450 cells/µL naive CD4+ T cells [49].
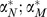	Excess activation of resting naive ( 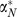 ) and resting memory cells ( 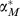 ) due to HIV (correlates with total viral load, see Methods)	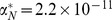 1/day/virion; 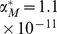 1/day/virion	Determined to give a decline of total CD4+ T cells from 770 cells/µL at end of PHI to 200 cells/µL at 10 years post-PHI [50].
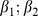	Parameters determining number of activated naive CD4+ T cells in PB. Here 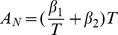	β_1_ = 10×(5×10^6^) cells; β_2_ = -0.0095	From [21]. Results in increasing numbers of activated naive CD4+ T cells with decreasing total CD4+ T cell number 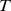 in PB. Gives approx. 1 cell/µL of activated naive CD4+ T cells at total CD4+ T cell count of 1000 cells/µL. Increases to approximately 10 cells/µL at total CD4+ T cell count of 100 cells/µL.
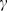	Parameter determining clonal expansion following cell activation	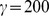	From [68], [69]. Assumes that a cell undergoes 5 to 10 mitiotic cycles during division following activation. The choice 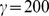 assumes approximately 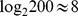 mitiotic cycles following activation.
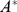	Setpoint determining number of activated cells	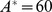 cells/µL	From [40].
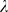	Deactivation rate of activated cells, resulting in new memory cells	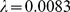 1/day	From [40], [68]. Here 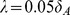 . Assumes that 95% of activated CD4+ T cells die, with 5% reverting to memory.
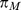	Homeostatic proliferation rate of resting memory cells	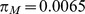 1/day	From [67].
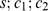	Parameters describing thymic export rate in healthy individual. Thymic export rate is given by 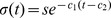	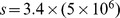 cells/day; 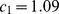 1/day; 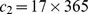 days	From [37]. Results in supply of approx. 2 cells/µL/day at age 30, which declines to approx. 1.3 cells/µL/day at age 40.
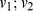	Parameters describing homeostatic proliferation rate of resting naive cells given by 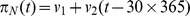 . Assumed to increase with age to compensate for declining thymic export with age	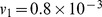 1/day; 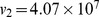 1/day/day	Determined to compensate for thymic decline with age, so that naive CD4+ T cell levels in a healthy individual remain constant [49].
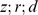	Parameters modelling reduction of thymopoiesis with duration of untreated infection [38] through the term 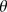 (here 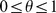 ) given by the equation 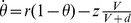 .	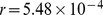 ; 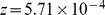 ; 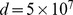 virions;	Calibrated to give naive CD4+ T cells increases of approximately 100 cells/µL over 2 years on HAART with 90% efficacy [70].

**Table 2.Table:** of model parameters relating to virion dynamics and dynamics of productively infected cells.

Parameter	Parameter description	Parameter values	Source
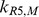 ; 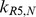 ; 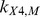 ; 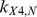 ; 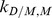 ; 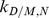	Infectivities of R5, D/M and X4 for the two target cell populations.Here 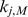 and 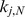 denote the infectivity of strain 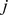 (where 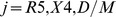 ) for activated memory ( 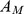 ) and for activated naive ( 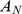 ) CD4+ T cells respectively	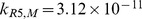 ; 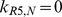 ; 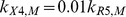 ; 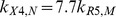 ; 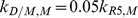 ; 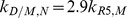 ;Units : 1/day/virion. N.B. : each infectivity (except 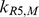 ) is expressed as a multiple of 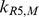	Determined by model calibration to give: 1.) R5 viral load setpoint of approximately 4.5 log10 HIVRNA copies/mL at age 30 (end of PHI); 2.) X4 emergence with median time of approximately 4 years post-PHI [15]. See Methods for justification of relative values of the infectivities to one another .
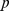	Production rate of new virions from productively infected cells	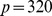 virions/cell/day	From [40,71].
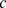	Virion clearance rate from body	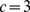 1/day	From [72].
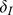	Death rate of productively infected cells	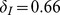 1/day	Gives half-life for productively infected cells of approximately 1.2 days [72].
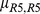 ; 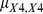 ; 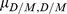 ;	Probability that no mutation from one viral strain to another occurs during the reverse transcription step of the infection	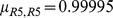 ; 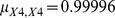 ; 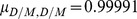	See Methods and Table 3.
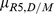 ; 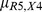 ; 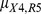 ; 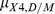 ; 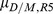 ; 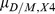	Probability that mutation from one viral strain to another occurs during the reverse transcription step of the infection. Here 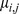 denotes the probability of mutation from strain 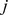 to strain 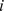 , where 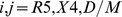 .	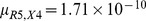 ; 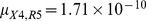 ; 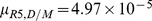 ; 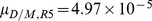 ; 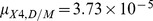 ; 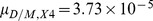	See Methods and Table 3. Here mutation from R5 to D/M is more likely than mutation from R5 to X4.
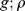	Parameters that determine viral growth rates and that model increasing viral infectivity over time (independently of viral tropism) via the term 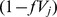 where 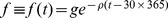	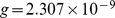 1/virion ; 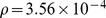 1/day	Determined to give : 1.) R5 viral setpoint of approximately 4.5 log10 HIV RNA copies/mL at age 30 which increases to 5 log10 HIV RNA copies/mL after 10 years of infection at age 40 ; 2.) Viral regrowth from approx. 50 HIV RNA copies/mL to approx. 4.5 log10 HIV RNA copies/mL in 4 weeks following cessation of HAART.
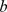	Parameter determining increases in infectivities of D/M and X4 for activated memory CD4+ T cells following suppression of R5 virus by CCR5 blockers. This is modelled via the terms 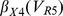 and 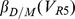 .	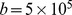 virions	Determined to give increased infectivity of D/M and X4 virus for activated memory CD4+ T cells once R5 virus drops below approximately 3 log 10 HIV RNA copies/mL (see Supplementary Figures S1 and S2).



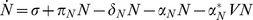


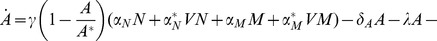


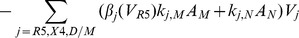


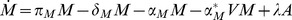


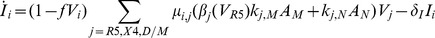


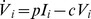
where dots above variables denote derivatives with respect to time. Here 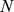, 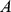 and *M* respectively denote the total number of resting naive, activated and resting memory CD4+ T cells at time 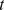 (in days) in peripheral blood (PB). All simulations were run from 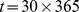
days (corresponding to age 30) until 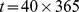 days (corresponding to age 40), with age 30 assumed to correspond to the end of primary HIV infection (PHI). When scaling from total numbers in PB to concentrations in PB, we assume a 5L PB volume. Although simulations for CD4+ T cells and HIV RNA copies are shown per µL and per mL of PB respectively, all calculations including mutations are determined over the entire 5L of PB.


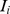
 and 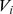 respectively denote the total number of productively infected cells of strain 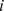 in PB and also the total number of viral particles of strain 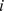 in PB. Here 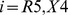 or 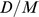 tropic virus. The effective viral population sizes in our simulations are taken as the total numbers of virions in PB.

The variables 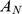 and *AM* respectively denote the number of activated naive CD4+ T cells (CD4+HLA-DR+CD45RA+) and activated memory CD4+ T cells (CD4+HLA-DR+CD45RO+) in PB. It is assumed in our model that only activated cells may give rise to productively infected cells upon infection [33], [34]. Here 
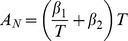
, where 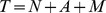 denotes the total number of CD4+ T cells in PB, with the parameters 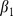 and 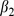 chosen so that 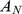 increases monotonically with decreasing total CD4+ T cell numbers 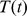 [21], [23], [24], reflecting increased numbers of activated naive CD4+ T cells (that only express CXCR4 [22], [35]) at later stages of the infection [21]. Activated memory CD4+ T cells (that predominately express CCR5, but also express CXCR4 [22], [35]) are assumed to be given by 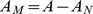. Since 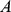 is always regulated to a setpoint of approximately 60 cells/µL (see below) and since 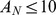 cells/µL at any timepoint [21], the number 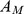 of activated memory CD4+ T cells is always guaranteed to be non-negative in our simulations.

The term 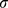 denotes the rate of thymic export of naive CD4+ T cells into PB. Here 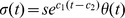, with the parameters 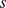, 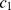 and 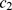 determined so that thymic output declines with age [36], [37]. The term 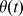 (here 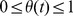) models reduction of thymopoiesis with duration of untreated infection [38]. It is assumed that 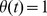 in a healthy individual. The term 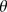 is governed by the equation 
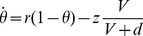
 (here 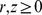 are parameters), so that presence of virus (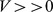) reduces thymic supply (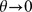), but suppression of virus (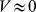) results in restoration of thymic supply (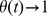). Here 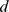 denotes a parameter so that viral loads of above approximately 4 log10 HIV RNA copies/mL result in decline of thymic production (when 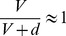), but values below result in slow restoration of thymic function (when 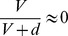).

Here 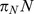 and 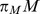 denote homeostatic proliferation of resting naive and resting memory CD4+ T cells respectively, with the rate of naive proliferation assumed time-varying and given by 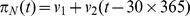, so that the homeostatic proliferation of resting naive cells increases monotonically with time, compensating for decline in thymic function with age. This ensures constant naive and memory CD4+ T cell numbers in a healthy individual.

In our model 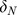, 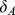, 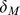 and 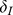 respectively denote death rates of resting naive, activated, resting memory and productively infected CD4+ T cells. 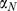 and 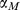 denote normal activation rates (in a healthy individual); 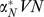 and 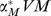 denote excess activation due to HIV, that is assumed to correlate with the total viral load 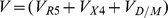 [39].

The term 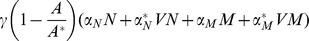 denotes clonal expansion following activation. Activated CD4+ T cells are assumed to expand by a factor of 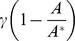, which results in the regulation of the total number of activated CD4+ T cells to the setpoint value of 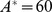 cells/µL as observed during HIV infection [40]. The term 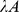 denotes reversion from the activated to the quiescent/resting state.

In the present model 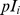 denotes production of new virions of strain 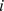, with 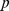 denoting the corresponding production rate of new virions by productively infected cells. It is assumed that the viral production rate does not differ across the three viral strains. In our model 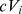 denotes clearance of free virions, with 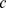 denoting the clearance rate.

The term 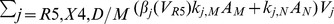 denotes the infection rate of activated CD4+ T cells over the three viral strains, whereas 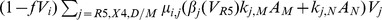 denotes the rate of increase of productively infected cells of strain 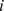. Productively infected cells of strain 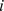 may arise as a result of infection by strain 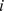 if no mutation to a different strain occurs during reverse transcription, or may also arise as a result of infection by strain 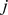 (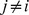) if a mutation from strain 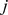 to strain 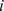 occurs. Furthermore:

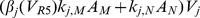
 denotes new infections by strain 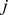. Here 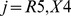 or 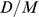,
- The parameters 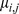 denote mutation rates from strain 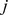 to strain 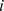 during the reverse transcription step of the infection cycle (see below for calculation of 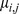). Here 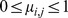 and 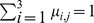. When 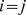 the rate 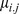 denotes the fraction of infections by strain 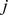 not resulting in mutation to a different strain during infection.

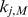
 and 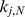 denote the infectivities of strain 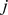 for activated memory (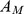) and for activated naive (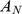) CD4+ T cells respectively. Since R5 and X4 exhibit increased fitness over D/M strains [32], we assume that 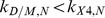 and 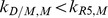. Since activated memory CD4+ T cells express CXCR4 [22], [35] we assume that both D/M and X4 virus can infect these cells, i.e. we assume 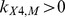 and 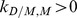. However, since activated memory CD4+ T cells predominately express CCR5 [22], [35], we also assume that 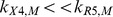, so that the infectivity of X4 for activated memory CD4+ T cells is smaller than the infectivity of R5 for activated memory CD4+ T cells. We also assume that X4 infection of activated memory CD4+ T cells is less efficient than X4 infection of activated naive CD4+ T cells, since activated memory CD4+ T cells exhibit lower CXCR4 expression than activated naive CD4+ T cells [22], [35], i.e. we assume 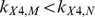. It is also assumed that R5 tropic virus does not infect activated naive CD4+ T cells, since these only express CXCR4 with negligible/no CCR5 expression [22], [35], so that 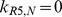,

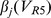
 are dimensionless quantities modelling increased infectivities of X4 and of D/M for activated memory CD4+ T cells when R5 is suppressed by CCR5 blockers [11], [12], [18]. In the above equations, 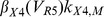 and 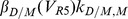 respectively denote the “*modulated infectivities*”(i.e. modulated by R5 viral load) of X4 and of D/M for activated memory CD4+ T cells. Since activated memory CD4+ T cells predominately express CCR5 [22], [35] (but also express CXCR4 [22], [35]), we assume that R5 presence inhibits X4 and D/M viruses, but that selection for X4 and D/M viruses increases following R5 suppression [11], [12], [18] as a result of increased availability (since R5 is suppressed) of CD4-CXCR4 complexes for D/M and X4 viral binding on activated memory CD4+ T cells. To model this effect, we assume (for 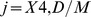) that 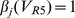 when 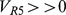, but that 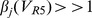 when 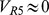. We let 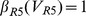 (i.e. trivially the R5 virus does not inhibit itself). Furthermore we let: 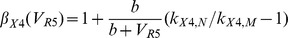


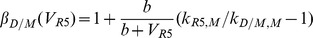
where 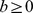 denotes a parameter. The plots of the modulated infectivites 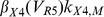 and 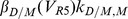 under these choices of 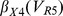 and 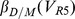 are shown in Figures S1 and S2. Our choice ensures that in the above equations 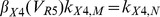 when 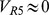, so that the *modulated infectivities* 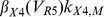 of X4 for activated memory CD4+ T cells increases (after R5 is suppressed) to equal the infectivity 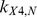
of X4 for activated naive CD4+ T cells. This amounts to an increase in infectivity since 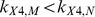 from previous dot-point above. The choice of 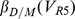 ensures that in the above equations 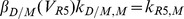 when 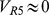, i.e. the *modulated infectivities* 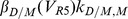 of D/M virus for activated memory CD4+ T cells increases (after R5 is suppressed) to equal to the infectivity 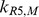 of R5 for activated memory CD4+ T cells. This amounts to an increase in infectivity, since 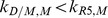 from previous dot-point above. These assumed increases in infectivites of D/M and X4 for activated memory CD4+ T cells, once R5 has been suppressed by CCR5 blockers, are most likely overconservative (see Discussion), but these worst-case assumptions are imposed to ensure robustness of the present analysis with regards to the likelihood of X4 selection following suppression of R5,
- The term 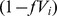 ensures that viral growth rates decrease at higher viral loads as the equilibrium viral setpoint is approached. Here 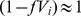 when 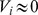, but 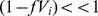 when 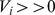, so that viral growth rates slow down as the viral setpoint is approached. To model increasing viral fitness and increasing viral loads over time occurring independently of viral tropism [41], [42], [43], we assume that 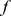 is time-varying, independent of whether treatment is received over the course of infection, and given by 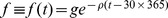 where 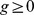 and 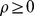 are parameters selected so that R5 tropic virus exhibits a viral load of approximately 4.5 log10 HIV RNA copies/mL at end of PHI (at age 30) which then increases to 5 log10 HIV RNA copies/mL after 10 years of untreated infection (by age 40). Plots of the term 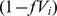 at various stages of infection and also as a function of 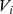
are shown in Figure S3. Our choice of 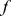 also ensures a viral load recrudescence from 50 HIV RNA copies/mL to approximately 4.5 log10 copies/mL over a period of approximately four weeks, in line with in-vivo observations following cessation of HAART [44], [45], [46],

### Calculation of Viral Mutation Rates 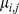


**Figure 2:**
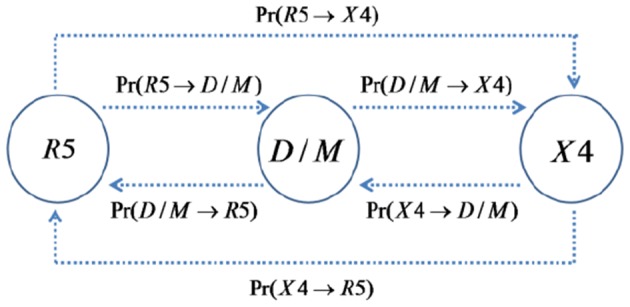
Three-state Markov model of viral strain mutations during the reverse transcription step of the infection cycle. There are three viral strains (R5, D/M,X4 as shown in circles) and a total of six possible mutations. Here 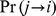 denotes the probability of transition/mutation from viral strain 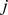 to viral strain 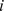 at the reverse transcription step of infection. The dashed lines denote transitions/mutations between viral strains. The probabilities of transition/mutation between different strains are calculated under the assumption that viral tropism is determined by the amino acids at positions 11, 25 and 29 in the V3 region of env (see Methods and Table 3).



For any HIV virion that enters a cell, it is assumed that viral mutations may occur during the reverse transcription step of infection (see above). It is assumed that the 3 amino acids at positions 11, 25 and 29 in the V3 region of the env gene determine viral tropism [28] as given in Table 3. The possible mutations between the three viral strains R5, D/M and X4 are given in Figure 2. The transition probability from viral strain 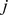 to viral strain 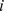 is calculated by summing over probabilities of all possible three letter amino acid mutations that give rise to the mutation from 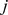 to 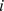:

**Table 3.Table:** of three-letter amino acid sequences that determine viral tropism.

Viral strain	Three-letter amino acid sequence corresponding to amino acids at positions 11,25 and 29 in V3 region of env
R5 tropic strain (R5)	SDD
X4 tropic strain (X4)	RQD, RDN, RQN, SRD
Dual/Mixed tropic (D/M)	Any other permutation of three-letter amino acid sequences

It is assumed that viral tropism is determined by the three amino acids at positions 11, 25 and 29 in the V3 region of env [28].

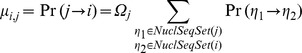
where 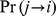 denotes the probability of mutation from viral strain 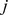 to viral strain 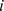. Here 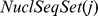 and 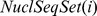 denote the sets of all possible nucleotide sequences corresponding to the viral strains 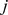 and 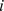 respectively. Each nucleotide sequence contains nine nucleotides corresponding to three codons associated with the three-letter amino acid sequence for each strain. The term 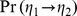 denotes the probability of mutation from nucleotide sequence 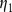
to nucleotide sequence 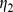. Assuming that mutations in the nucleotide sequence mutate independently of one another, then the probability 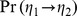 is calculated by the following:



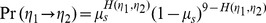
Here 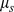 denotes the probability that one nucleotide base mutates into another nucleotide base. To calculate the average probability of mutation between each strain, it is assumed that the average probability of mutation for any nucleotide base is 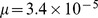 per replication cycle [26]. It is consequently assumed that mutation from any nucleotide is equally likely to any of the other three nucleotides, so that 
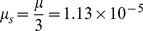
. Here 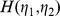 denotes the Hamming distance between nucleotide sequences 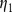 and 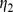, so that 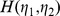 denotes the number of positions at which the nucleotide sequences differ (here 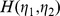 is an integer between 0 and 9).

The term 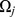 denotes a normalization factor required to ensure “well-defined” probabilities, i.e. so that 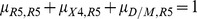, 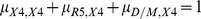 and 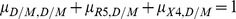. The normalization factor 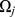 from above is defined by 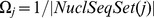, where 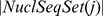 denotes the number of nucleotide sequences in the set 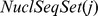.

### Initial Values for Model Variables

The course of infection is simulated from age 30 to age 40 (i.e. from 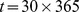 days to 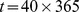 days). It is assumed that age 30 corresponds to the end of PHI, with a total CD4+ T cell count of 770 cells/µL. Since naive CD4+ T cells counts are generally lower during HIV infection, we initialize our simulation so that 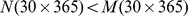 at age 30, with 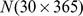 and 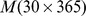 initialized to cell counts corresponding to concentrations of 240 and 470 cells/µL respectively. The initial number 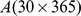 of activated CD4+ T cells was initialized to correspond to 60 cells/µL. All simulations were implemented and run in Mathworks MATLAB 2010b. The initial R5 viral load 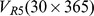 was initialized to a value corresponding to 4.5 log10 HIV RNA copies/mL and the initial number of productively infected cells 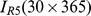 of R5 tropism was initialized to a value of 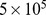 cells. Number of viral particles and number of productively infected cells for D/M and X4 were all initialized to zero, reflecting the assumption that initial infection occurs with an R5 virus. The term 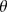 modelling decline of thymopoiesis is initialized to 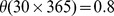, so that an individual at end of PHI (age 30 in our simulations) has a 20% reduction in thymopoesis compared to a healthy individual. The time of X4 emergence is defined as the time at which the X4 viral load exceeds a value of 100 HIV RNA copies/mL.

### Solution of Model Equations and Modelling of Viral Stochastic Effects at Low Population Densities

The deterministic differential equations from above describe the system evolution when population sizes are “large”. However when populations sizes become “small”, a birth/death stochastic equation model is used to model stochastic drifts at small population sizes [47]. In particular, given any time-dependent variable 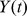 from above describing evolution over time, we first write the corresponding differential equation as follows:
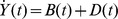
where 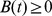 denotes the sum of all “birth” terms (all positive terms in the differential equation for 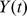) and where 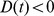 denotes the sum of all “death” terms (all negative terms in the differential equation for 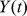). The differential equation is then solved via Euler’s method with a stepsize of 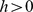:



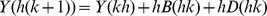
where 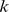 denotes the discrete index of the difference equation. The above difference equation is employed for values above limit 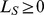 (i.e. if 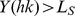). For values below the limit (i.e. for 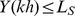), the following stochastic equation (modelling stochastic drifts at small population sizes) is employed:



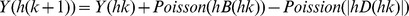
where 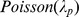 denotes a random value drawn from the Poisson distribution 
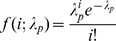
 with event occurrence rate 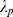, and where 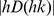 denotes the absolute value of 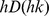 (since 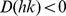). We select the stochastic limit as 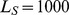 over all 5L of PB, which equates to a limit of 0.2 virions per mL, which is a reasonable value given that assessment of HIV RNA with single copy assays indicates that HIV RNA settles to a median of 9 copies per mL under antiretroviral therapy [48]. We also employ a time stepsize of 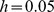 days for the discretized equations.

### Determination of Model Parameters

Model parameters are given in Tables 1 and 2, and were selected from the literature, with unknown model parameters determined by model calibration against known in-vivo dynamics of CD4+ T cells and viral loads. The model was calibrated to reflect the following dynamical aspects of the in-vivo biology:

1. Constant total CD4+ T cell count of approximately 1000 cells/µL in a healthy individual, with 500 cells/µL resting memory CD4+ T cells and 450 cells/µL resting naive CD4+ T cells [49],
2. Decline from approximately 750 cells/µL at the end of PHI (at age 30) to below 200 cells/µL in 10 years (by age 40) for an individual infected with R5-tropic virus, in whom no D/M and/or X4 tropic virus emerges [50]. R5 viral load of approximately 4.5 log10 HIV RNA copies/mL that increases to 5 log10 HIV RNA copies/mL after 10 years of untreated infection, reflecting increasing viral fitness (that is independent of viral tropism) with duration of infection [41], [42],
3. Viral recrudescence from below detection (50 HIV RNA copies/mL) to approximately 4.5 log10 HIV RNA copies/mL after 4 weeks following cessation of HAART [44], [45], [46],
4. Median time of X4 emergence of approximately 4 years post-PHI [15], resulting in AIDS (<200 cells/µL) within approximately 1–1.5 years following X4 emergence [13].

### Reduction in Viral Infectivity under Maraviroc and under BT

Administration of BT is assumed to multiply the infectivities of each of the above strains by 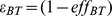, where 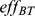 denotes the efficacy of BT. Administration of maraviroc is assumed to multiply the infectivity of only the R5-tropic strain by 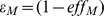, where 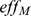 denotes the efficacy of maraviroc. Here maraviroc is assumed *not to inhibit* D/M strains in order to consider the worst-case scenario in the present analysis, despite previous studies reporting that maraviroc may inhibit some D/M strains [51]. If both BT and maraviroc are applied together, then the infectivity of R5-tropic virus is multiplied by a factor of 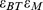, reflecting independent effects of the combined therapy. Maraviroc is also assumed *not to inhibit* X4 viral strains, since these utilize the CXCR4 coreceptor that is not blocked by maraviroc. It is assumed in all simulations that maraviroc has an efficacy of 90% (i.e. 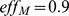).

## Results

### Model Simulation for an Untreated Individual Initially Infected with R5 Virus and in Whom no Viral Mutations Occur

**Figure 3:**
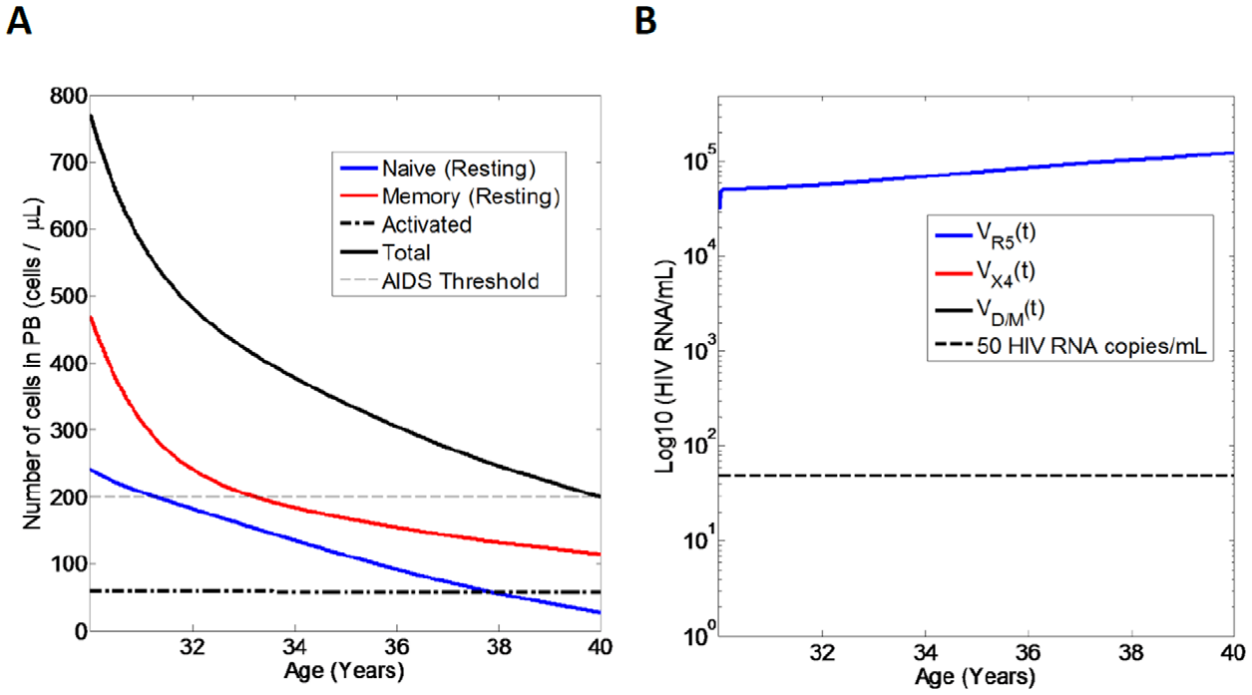
Model simulation for an untreated HIV-infected individual initially infected with R5-tropic virus, assuming no mutations. Age 30 denotes end of PHI and the simulation is run until age 40. A) CD4+ T cell numbers (cells/µL) in peripheral blood. Shown are numbers of naive resting (blue line), memory resting (red line), number of activated CD4+ T cells (black dash-dot line) and also the total number of CD4+ T cells (black line). Also shown is the AIDS threshold (200 cells/µL, dashed line); B) HIV RNA copies/mL in peripheral blood. HIV RNA for R5 tropic virus (blue line). Also shown is the 50 HIV RNA copies/mL limit of detection (black dashed line).

To validate the model, we first simulate the course of untreated HIV infection (Figure 3) where an individual is infected with R5-tropic virus and in whom no mutations to D/M or X4 virus occur (N.B. all subsequent simulations however assume inclusion of viral mutations in the model). Here the total CD4+ T cell counts decline from a value of 770 cells/µL at age 30 to a value below 200 cells/µL (AIDS) after approximately 10 years at age 40 in line with the clinical course of untreated HIV infection with R5 tropic virus [50]. Resting naive CD4+ T cells exhibit lower counts than resting memory CD4+ T cell counts in line with clinical observations [52]. Activated CD4+ T cells exhibit a value of 60 cells/µL throughout the course of infection [40]. Over the course of infection, the viral load exhibits an increase from a value of approximately 4.5 log HIV RNA copies/mL at age 30 to a value of 5 log HIV RNA copies/mL after 10 years of infection by age 40, reflecting increasing viral fitness/diversity over the course of infection that is independent of viral tropism [41], [42], [43].

### Model Simulation for an Untreated Individual Initially Infected with R5 Including Viral Mutations

**Figure 4:**
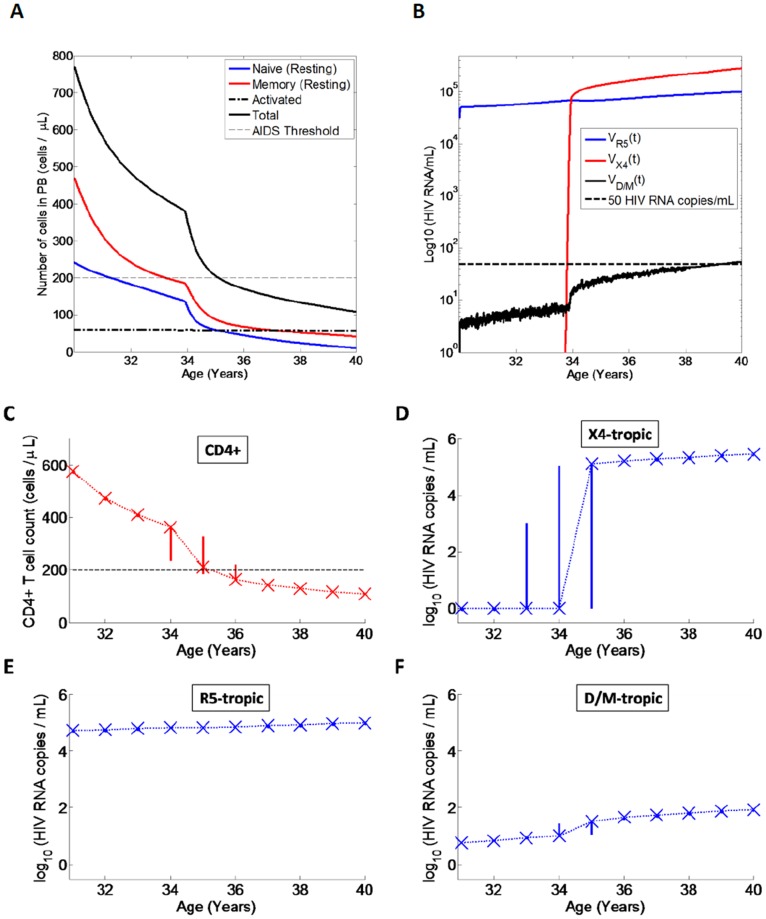
Untreated HIV infection in individuals initially infected with R5-tropic virus. Shown are a single simulation and the Monte Carlo simulations. A) CD4+ T cell counts and B) HIV viral load for a single simulation in which X4 emerges approximately 4 years post-PHI; C) Total CD4+ T cell counts, D) X4 tropic viral load, E) R5 tropic viral load and F) D/M tropic viral load for Monte Carlo simulations (250 trials) of untreated HIV infection. Simulation outcomes were stored at 1 year intervals from year 1 to year 10 post-PHI. Crosses denote medians at each time-point. The solid vertical lines at each time-point denote interquartile ranges. Dotted lines connect medians across different time-points. The dashed black line in Panel C denotes the AIDS threshold (200 cells/µL). In the plots of viral loads, any viral load values below 1 HIV RNA copy/mL were set to 1 HIV RNA copy/mL.

Next we simulate an untreated individual initially infected with R5-tropic virus but now (and hereafter) assuming viral mutations in our model. A single simulation for an individual in whom X4 emerges at approximately 4 years post-PHI is shown in Figure 4 A, B. Unlike the course of infection with only R5 tropic virus (Figure 3 A), here the CD4+ T cell counts exhibit an increased decline rate following emergence of X4 tropic virus (Figure 4 A), resulting in accelerated progression to AIDS [13], [15]. Our model results in progression to AIDS within 1–1.5 years following emergence of X4-tropic virus in line with clinical observations for the case that X4 virus emerges when CD4+ T cell counts exhibit a value of approximately 400 cells/µL [13]. Furthermore, both R5 and X4 tropic viruses coexist following the switch (Figure 4 B) in line with clinical observations [15]. Throughout the course of infection (including prior to the X4 switch), D/M topic viral strains exhibit viral loads between 1 and 50 HIV RNA copies/mL (Figure 4 B). However, as a result of lower fitness (relative to R5 and X4 tropic virus; see Methods), D/M strains do not increase above 50 HIV RNA copies/mL throughout the course of infection.

**Figure 5:**
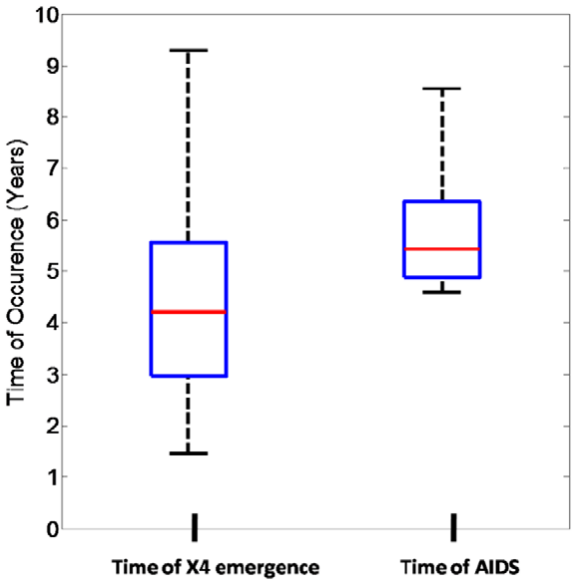
Times of X4 emergence and times of AIDS for the Monte Carlo simulations (250 trials) from Figure 4 C,D,E,F. Red horizontal bars denote medians. Boxes denote interquartile ranges. Whiskers denote the 5^th^ and 95^th^ percentiles. X4 emergence occurs with a median time of approximately 4 years post-PHI, resulting in AIDS with a median time of approximately 5.5 years in line with clinically observed courses of infection in which X4 emergence is observed.

We also performed Monte Carlo sampling over 250 trials for the case of untreated infection (Figure 4 C,D,E,F). Emergence of X4 virus occurred with a median time of approximately 4 years post-PHI (Figure 5), followed by accelerated progression to AIDS with a median time of AIDS of approximately 5.5 years post-PHI in line with clinical observations. Our stochastic model also captured variation in the time of X4 emergence around the median time of 4 years post-PHI (Figure 5), with X4 emergence also occurring earlier than 4 years post-PHI [20].

### Application of Maraviroc as Monotherapy without BT

**Figure 6:**
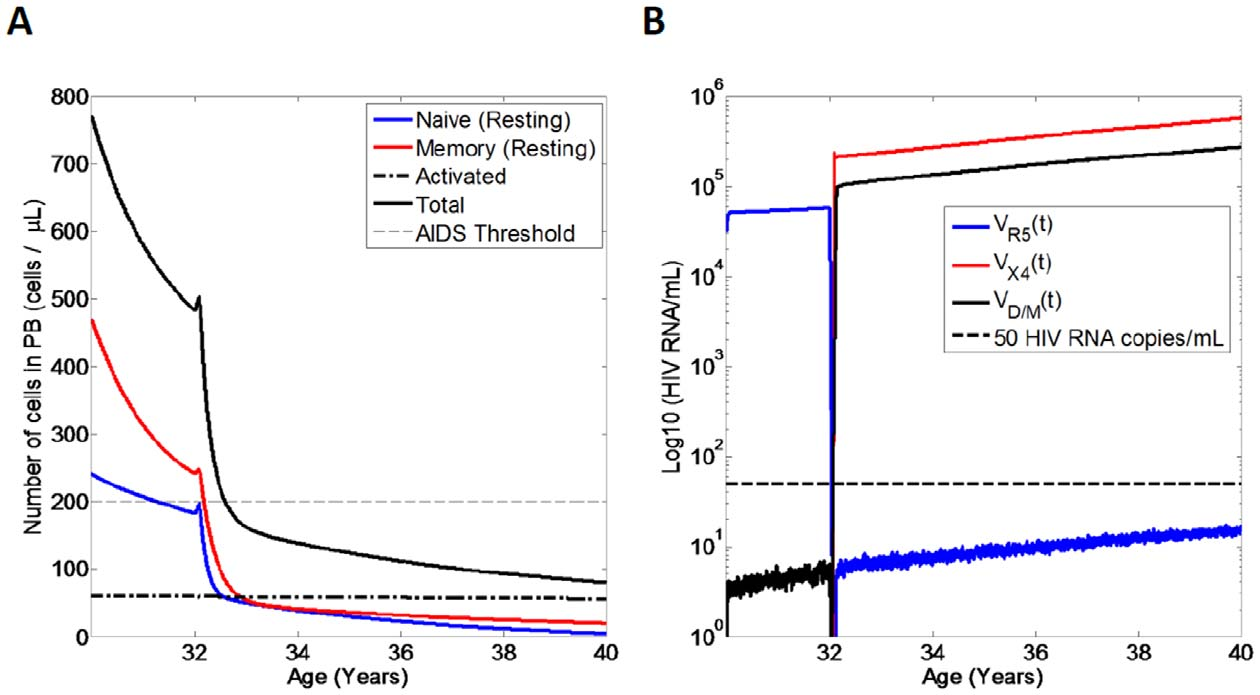
Simulation of an HIV infected individual who receives maraviroc as a monotherapy (i.e. BT has 0% efficacy). Shown are the CD4+ T cell counts and the viral loads over time. A) CD4+ T cell counts and B) Viral loads for an individual who receives maraviroc (90% efficacy) as a monotherapy without any BT after 2 years post-PHI (i.e. after age 32).

Next we investigated whether administration of maraviroc without BT (i.e. BT has efficacy 0%) would promote selection for X4 tropic virus and result in accelerated progression to AIDS. To this aim, we simulated an individual who receives maraviroc as monotherapy at age 32 (Figure 6 A,B). Administration of maraviroc resulted in decay of R5 tropic virus (Figure 6 B) to below detection. In correspondence to the decay of R5 tropic virus, we observed selection for X4 and D/M tropic viral strains as a result of increased availability of CXCR4-CD4 complexes (on activated memory CD4+ T cells) due to decreased competition with R5 virus for activated memory CD4+ T cells (see Methods).

Following selection for X4 tropic virus after administration of maraviroc as monotherapy, total CD4+ T cell exhibit a rapid progression to AIDS within 1 year (Figure 6 A), so that AIDS occurs around the age of 33. These results illustrate that administration of maraviroc as a monotherapy can result in accelerated progression to AIDS as a result of selection for X4 tropic viral strains following suppression of R5 tropic virus. These results are in agreement with previous *deterministic* modelling [23], [24].

### Monte Carlo Simulations of Maraviroc Administration with BT of Moderate/High Efficacy

To further investigate the likelihood of selection for D/M and/or X4 tropic virus following administration of maraviroc, we performed Monte Carlo simulations of maraviroc administration at early, intermediate and late stages of infection respectively corresponding to therapy administration after 1, 3.5 and 6 years post-PHI. We considered scenarios in which maraviroc was applied with BT of moderate efficacy (BT has 90% efficacy) and also with BT of high efficacy (BT has 99% efficacy). Maraviroc was assumed to have an efficacy of 90% in all scenarios.

**Figure 7:**
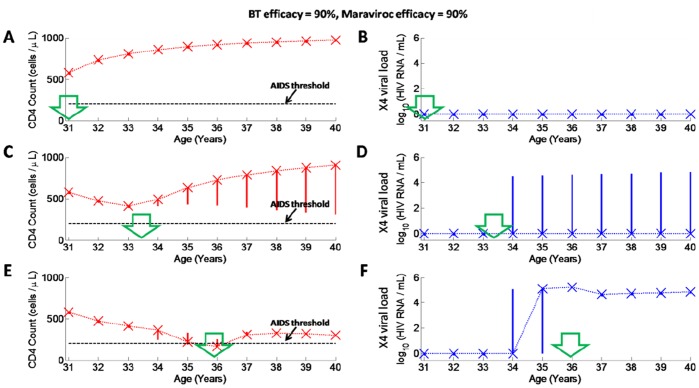
Monte Carlo simulations (250 trials) in HIV-infected individuals who receive maraviroc (90% efficacy) with BT of 90% efficacy at early, intermediate or late stages of infection. The green arrows pointing downwards in each plot indicate time when therapy was applied (at 1, 3.5 or 6 years post-PHI, corresponding respectively to age 31, 33.5 and 36). Any viral load values below 1 HIV RNA copy/mL were set to 1 HIV RNA copy/mL. Simulation outcomes were stored at 1 year intervals from year 1 to year 10 post-PHI. Crosses denote medians at each time-point. The solid vertical lines at each time-point denote interquartile ranges. Dotted lines connect medians across different time-points. The black dashed horizontal line in plots of CD4+ T cell counts denotes the AIDS threshold (200 cells/µL); A) Total CD4+ T cell numbers and B) X4 viral load for therapy administered at 1 year post-PHI; C) Total CD4+ T cell numbers and D) X4 viral load for therapy administered at 3.5 years post-PHI; E) Total CD4+ T cell numbers and F) X4 viral load for therapy administered at 6 years post-PHI.

First we investigated whether BT therapy of moderate efficacy (BT has 90% efficacy) would ensure sufficient X4 suppression (Figure 7). When this therapy is applied, then in the majority of patients no X4 selection is observed if therapy is applied at the early stage of the infection (Figure 7 A,B). However if therapy is applied at intermediate or late stages of infection, then X4 selection is still observed in a subset of patients (Figure 7 C,D,E,F), indicating that BT therapy of 90% efficacy is not sufficient to fully suppress X4 viral strains in all patients.

**Figure 8:**
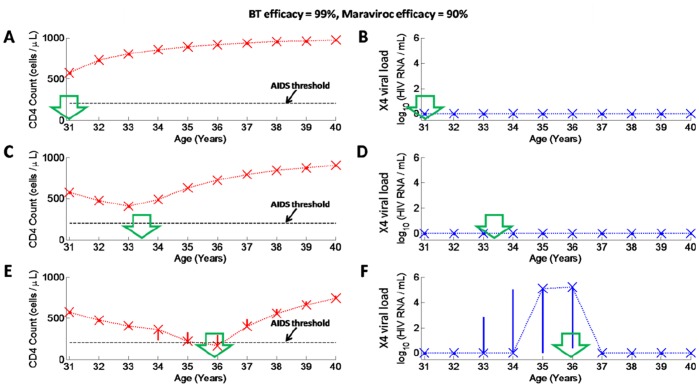
Monte Carlo simulations (250 trials) in HIV-infected individuals who receive maraviroc (90% efficacy) with BT of 99% efficacy at early, intermediate or late stages of infection. The legend for this figure is the same as for Figure 7.

Next we ran Monte Carlo simulations for the case that maraviroc is administered with BT of high efficacy (BT has 99% efficacy) as shown in Figure 8. Therapy was again assumed to be administered at early, intermediate and late stages of the infection. Of the 250 simulations not a single case of X4 emergence was observed when therapy was applied at any stage of infection, with all patients experiencing increases in CD4+ T cell counts under this therapy (Figure 8). Even if X4 emergence was observed prior to administration of therapy (Figure 8 F), X4 suppression was still observed following administration of therapy in all patients.

## Discussion

Here we investigated a *stochastic* model of X4 emergence to examine whether administration of CCR5 blockers in-vivo is likely to promote X4 emergence, thereby resulting in accelerated progression to AIDS. While previous studies investigated *deterministic* models of X4 dynamics where X4 emergence was driven by increasing activation of naive CD4+ T cells that express CXCR4 but not CCR5 [21], [23], [24], in the present analysis we extend upon these results by providing the first *stochastic* model of X4 emergence that explicitly includes CD4+ T cell activation dynamics. In addition to increasing activation of naive CD4+ T cells modelled previously [21], [23], [24], we also assumed that the tropism shift from R5 to X4 is driven by random viral mutations [28] of amino acids at positions 11, 25 and 29 in the V3 region of the env gene [27], [28], [29], [30], [31] (Table 3), which are strongly associated with viral tropism. Since X4 emergence is a *stochastic* event, with a median emergence time of approximately 4 years post-infection and a considerable variation in the time when X4 emerges [15], the model presented here is consequently more likely to be clinically relevant than previously presented *deterministic* models.

The key finding of the present analysis is that administration of maraviroc (90% efficacy) is safe at early, intermediate and also late stages of infection if administered with sufficiently potent BT (99% efficacy). Monte Carlo simulations of this scenario in the present analysis did *not* result in a single instance of X4 emergence (see Results). These results are encouraging given that X4 tropism is associated with accelerated progression to AIDS [13], [14], [15]. Our results are also in agreement with recent reports from the MOTIVATE clinical trials of maraviroc in patients with only R5 tropic virus, where maraviroc administered with OBT was reported *not to promote* X4 emergence [11], [12]. While D/M tropism was reported in those trials in approximately 50% of patients who experienced virological failure [11], [12], no CD4+ T cell decreases were reported in these individuals, indicating that selection for highly pathogenic X4 viral strains appears unlikely. Collectively therefore, the results of the present analysis, in line with reports from the MOTIVATE trials [8], [9], [10], [11], [12], indicate that in-vivo administration of maraviroc with BT of sufficient potency is safe and not likely to promote in-vivo selection of highly pathogenic X4 viral strains in patients with only R5 virus.

The results of the present analysis are further supported by observations that the virus can find novel ways to bind the CCR5 chemokine coreceptor when CCR5 blockers are administered in-vivo, rather than resorting to a tropism switch [1], [53], [54]. One possible explanation as to why the X4 switch is not likely to occur in this scenario is that most mutations in the V3 loop appear to result in a fitness disadvantage (i.e. mutation to D/M) rather than a tropism switch to X4 [55], with D/M virus less fit/pathogenic than pure X4 virus [32]. These observations indicate that the tropism switch to X4 may not necessarily be the most likely/efficient viral escape strategy when CCR5 blockers are administered in-vivo.

Our analysis also indicates that the in-vivo administration of CCR5 blockers as monotherapy (or with BT of suboptimal efficacy) can promote X4 emergence, thereby resulting in accelerated progression to AIDS. These results are in agreement with previous reports from three macaques dually infected with R5 and X4 tropic virus, where administration of the CCR5 blocker CMPD 167 as monotherapy was observed to promote outgrowth of X4 tropic virus [18]. Whether such observations also hold in human subject is uncertain, given that in-vivo administration of maraviroc as monotherapy in human subject has not been performed over an extensive time period to-date due to obvious resistance concerns.

The present conclusion, regarding safety of maraviroc with sufficiently potent BT, is likely to be very robust. We assumed that the infectivities of D/M and of X4 (for activated memory CD4+ T cells) increase following suppression of R5 virus, thereby resulting in increased selection for D/M and X4 viral strains [18]. This modelling choice was imposed to model increased availability of CD4-CXCR4 complexes for X4 and D/M viral binding on activated memory CD4+ T cells (following R5 suppression by maraviroc), as activated memory CD4+ T cells predominately express CCR5 but also express CXCR4 [22]. Since the actual increases in these infectivities are unknown, we assumed that: 1.) following R5 suppression, the infectivity of X4 for activated memory CD4+ T cells is equal to the infectivity of X4 for activated naive CD4+ T cells; 2.) following R5 suppression, the infectivity of D/M virus for activated memory CD4+ T cells is equal to the infectivity of R5 virus for activated memory CD4+ T cells. The first assumption is likely to be overconservative, given that per-cell CXCR4 density on activated memory CD4+ T cells is lower than on naive CD4+ T cells [22], so that X4 infection of activated memory CD4+ T cells should not be as efficient as X4 infection of activated naive CD4+ T cells. The second assumption is also likely to be overconservative, since D/M virus has been reported to be of lower fitness than R5 virus [32], [56], so that infection of activated memory CD4+ T cells by D/M virus should not be as efficient as infection by R5 virus. In addition, we also assumed that maraviroc does *not* inhibit D/M virus, whereas previous studies reported inhibition of some D/M strains by maraviroc [51]. Collectively, these represent worst case assumptions, so that the conclusions of the present analysis relating to X4 selection should be highly robust.

We extend on previous modelling by assuming that mutation from R5 to X4 occurs via intermediates that are of D/M tropism and that are of lower fitness than R5 and X4 [32], [57]. While stochastic mutations have previously been shown to account for X4 emergence via less fit intermediate viral strains [58], this previous modelling did not take into account shifting selective pressures driven by increased numbers of CXCR4-expressing naive CD4+ T cells at later stages of infection as in the present analysis. Furthermore, previous modelling only considered the application of CCR5 blockers at a single time-point [23], [24], whereas in the present case we consider the administration of maraviroc with BT at early, intermediate and late stages of the infection.

The model presented here has a number of limitations. Firstly, our modelling did not include additional cell populations such as macrophages that might contribute to increased selection for X4 virus at later stages of the infection when total CD4+ T cell counts are low, since macrophages provide an additional source of CXCR4-CD4 complexes [35], [59], [60]. However, given that macrophages exhibit lower levels of CD4 expression than CD4+ T cells [35], [61], [62], the omission of macrophages in our model should not alter the conclusion of the present analysis significantly. Secondly, in the present model only PB dynamics were modelled, whereas viral dynamics in lymph tissue are also likely to contribute to in-vivo selection for X4 viral strains. The thymus and bone marrow are likely to play a role in X4 selection and emergence [63], given that CD34+ hematopoietic stem cells express CXCR4 [64]. However, given the current lack of quantitative data regarding X4 dynamics in lymph tissue, this effect was not modelled in the present case. Thirdly, mutations outside positions 11,25 and 29 in the V3 region of the env gene are also known to determine viral tropism. However, the present model should still provide a reasonable approximation to actual in-vivo mutation dynamics, since in the present model most mutations in the V3 loop result in a fitness disadvantage rather than a tropism switch to X4 [55], with the virus more likely to mutate from R5 to D/M rather than to mutate from R5 to X4.

In conclusion, we have presented the first stochastic model of in-vivo X4 selection dynamics and investigated whether CCR5 blockers promote X4 emergence resulting in accelerated progression to AIDS. Since X4 emergence is a stochastic event with a median emergence time of approximately 4 years post-infection, and with considerable variation in the time when X4 emerges, the modelling results presented here are more likely to reflect likely clinical outcomes than previously presented *deterministic* models of X4 selection dynamics. The key result of our analysis and modelling is that the administration of maraviroc is safe and that it does not promote X4 emergence if employed with BT of sufficient potency. Our results also indicate that selection for X4 tropic virus may occur if BT of insufficient efficacy is administered with maraviroc. These results highlight the need for careful selection of BT when CCR5 blockers are administered in-vivo, as well as raise the question whether alternative anti-HIV CCR5-targeting treatments like gene therapy [65], [66] are more or less likely to promote selection of highly pathogenic X4 viral strains.

## Supporting Information

Figure S1
**Plot of the “modulated infectivity” 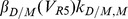 of D/M virus for activated memory CD4+ T cells as a function of R5 viral load 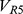.** Here 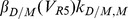 increases with lower R5 viral loads.(TIF)

Figure S2
**Plot of the “modulated infectivity” 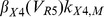 of X4 virus for activated memory CD4+ T cells as a function of R5 viral load 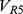.** Here 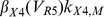 increases with lower R5 viral loads.(TIF)

Figure S3
**Plots of the term 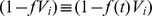 as a function of 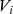 for 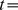
30×365, 32.5×365, 35×365, 37.5×365, 40×365 (corresponding respectively to age 30, 32.5, 35, 37.5 and 40 respectively).** Negative values are not shown above. The function values 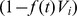 increase at later times 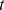, and decrease with higher values of 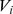. Here 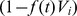 start to decrease significantly as 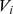 approaches approximately 4.5 log10 HIV RNA copies/mL, so that overall viral growth rates in our model start to slow significantly around 4.5 log10 HIV RNA copies/mL.(TIF)
