# Supplementary figures and images for: Stochastic Model of In-Vivo X4 Emergence during HIV Infection: Implications for the CCR5 Inhibitor Maraviroc
Source: PLoS One. 2012 Jul 17;7(7):e38755. doi: 10.1371/journal.pone.0038755 (PMC3398969; doi:10.1371/journal.pone.0038755)

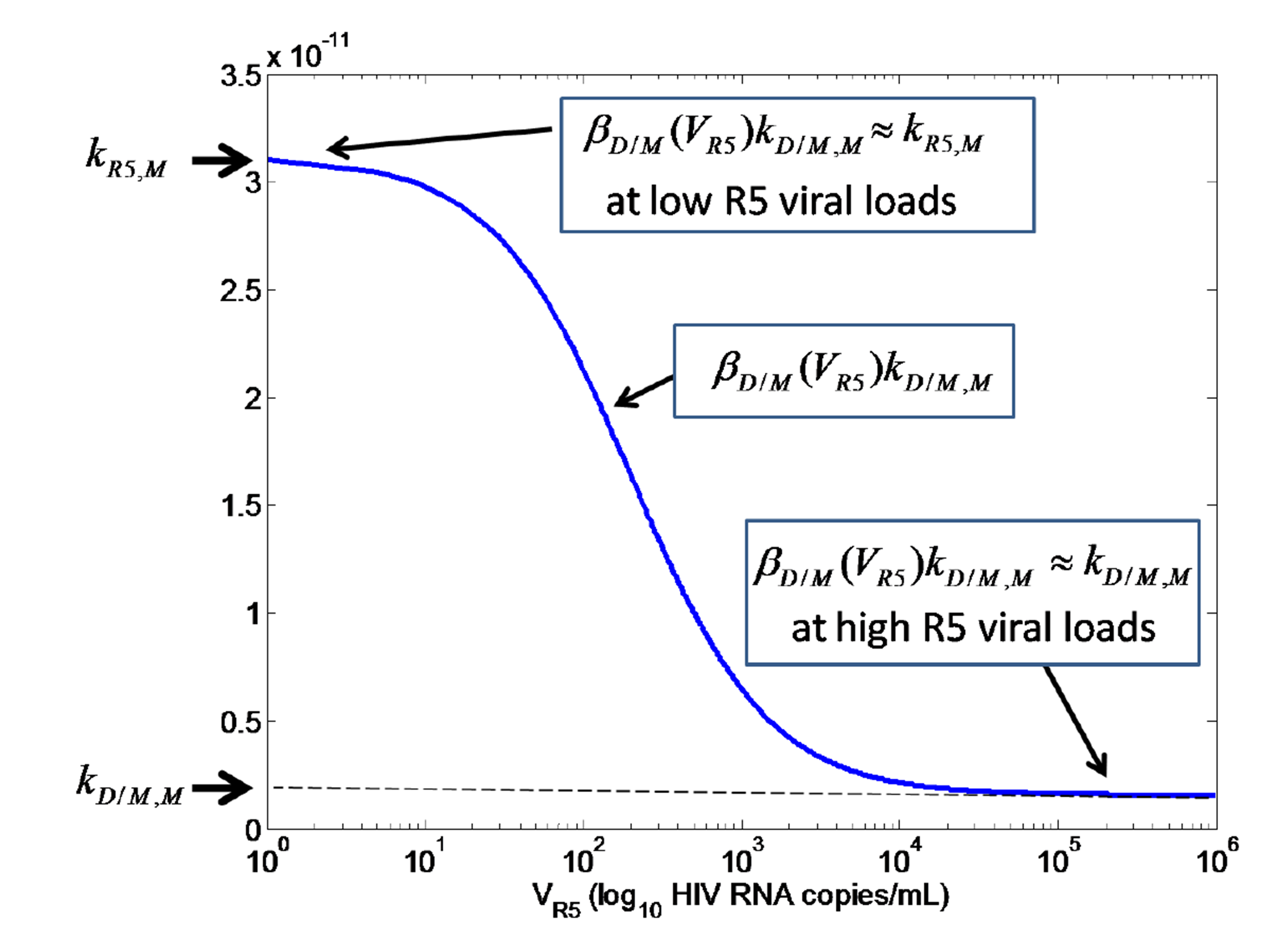

Supplement: Figure S1 — Plot of the “modulated infectivity” of D/M virus for activated memory CD4+ T cells as a function of R5 viral load . Here increases with lower R5 viral loads. (TIF) [file pone.0038755.s001.tif]

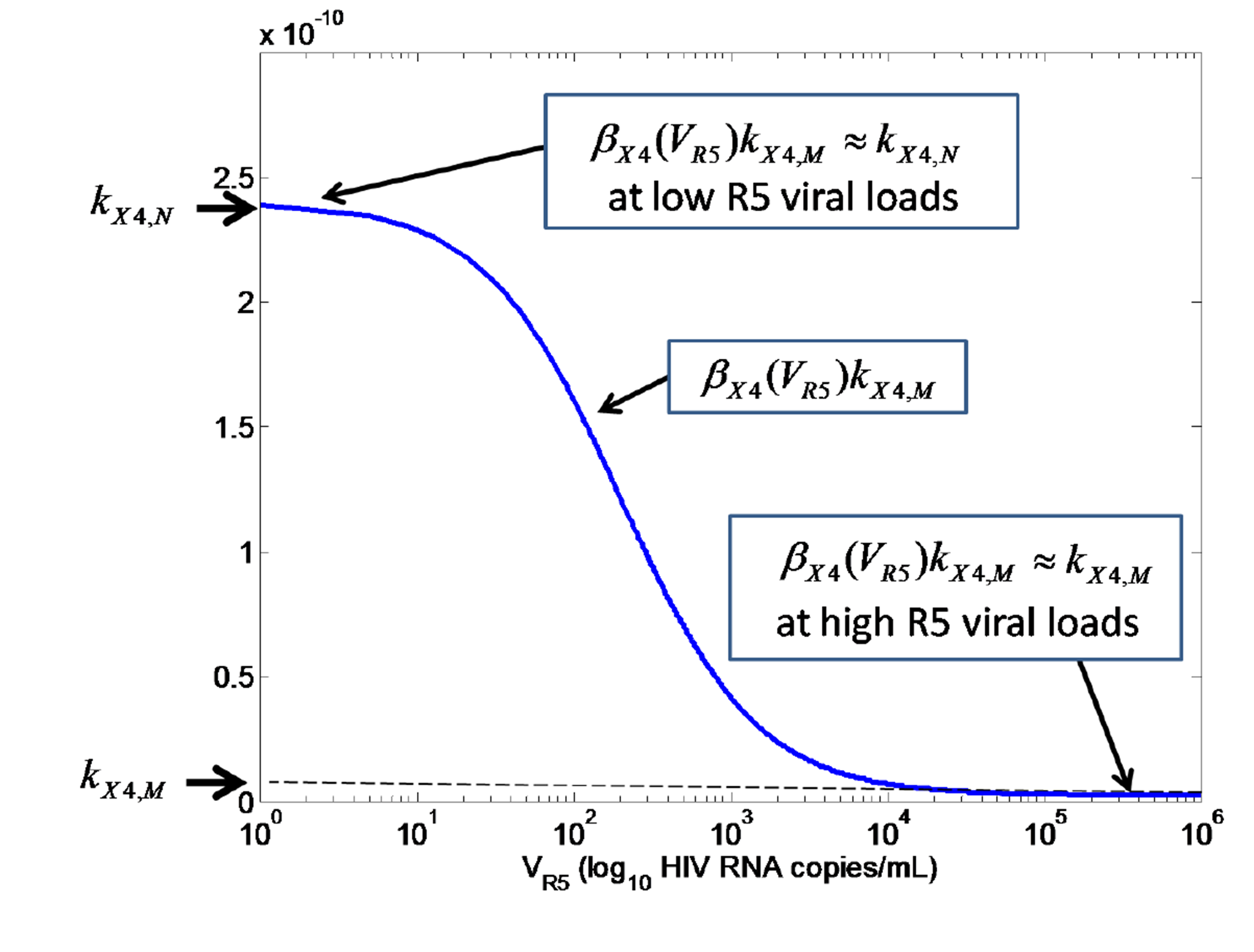

Supplement: Figure S2 — Plot of the “modulated infectivity” of X4 virus for activated memory CD4+ T cells as a function of R5 viral load . Here increases with lower R5 viral loads. (TIF) [file pone.0038755.s002.tif]

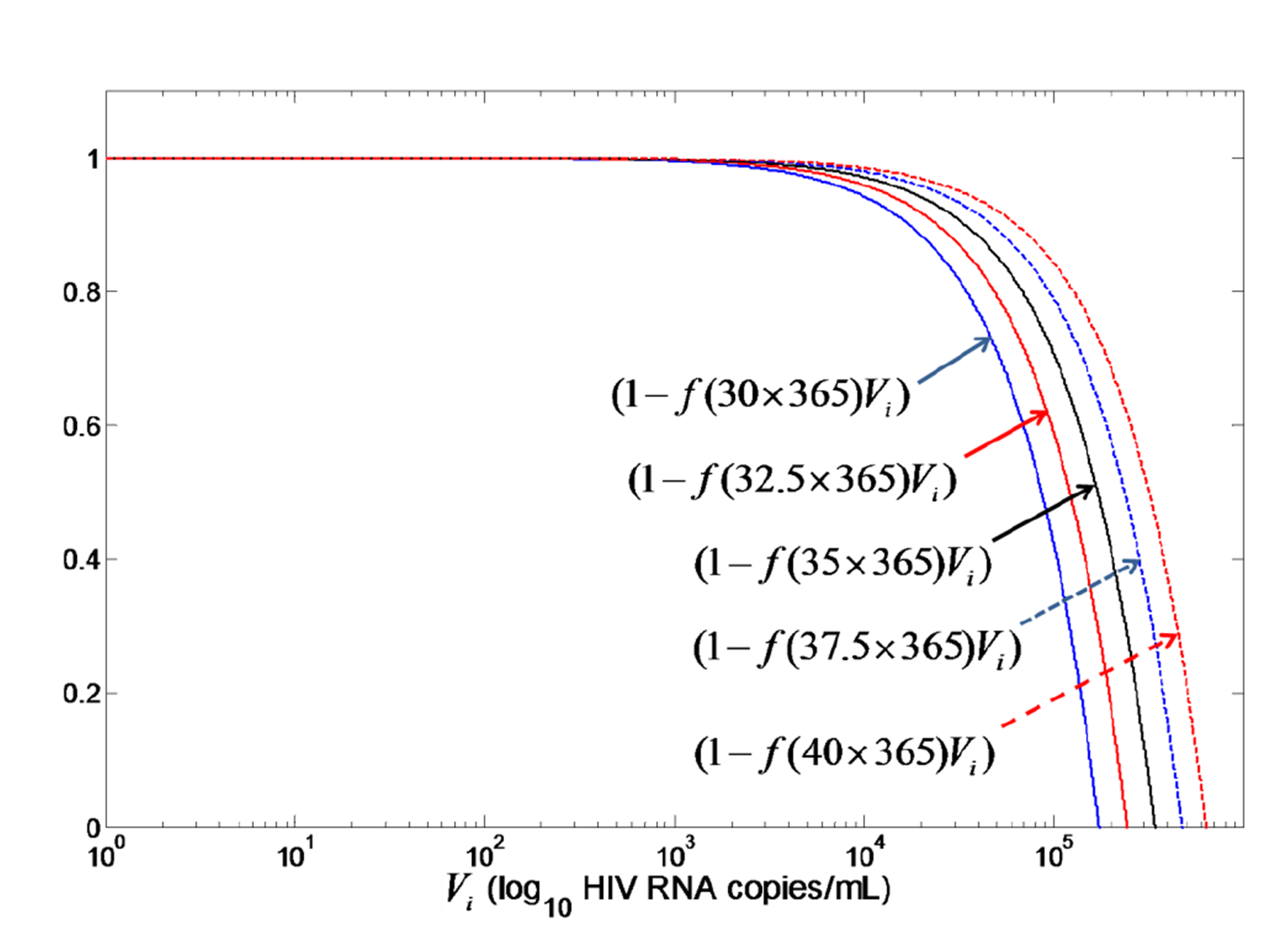

Supplement: Figure S3 — Plots of the term as a function of for 30×365, 32.5×365, 35×365, 37.5×365, 40×365 (corresponding respectively to age 30, 32.5, 35, 37.5 and 40 respectively). Negative values are not shown above. The function values increase at later times , and decrease with higher values of . Here start to decrease significantly as approaches approximately 4.5 log10 HIV RNA copies/mL, so that overall viral growth rates in our model start to slow significantly around 4.5 log10 HIV RNA copies/mL. (TIF) [file pone.0038755.s003.tif]
